# Time-lapse Raman imaging of osteoblast differentiation

**DOI:** 10.1038/srep12529

**Published:** 2015-07-27

**Authors:** Aya Hashimoto, Yoshinori Yamaguchi, Liang-da Chiu, Chiaki Morimoto, Katsumasa Fujita, Masahide Takedachi, Satoshi Kawata, Shinya Murakami, Eiichi Tamiya

**Affiliations:** 1 Department of Applied Physics, Graduate School of Engineering, Osaka University, 2-1 Yamadaoka, Suita, Osaka, 565-0871, Japan; 2 Institute of Photonics and Biomedicine, Graduate School of Science, East China University of Science and Technology, 130 Meilong Rd., Shanghai, 200237, China; 3 Department of Periodontology, Graduate School of Dentistry, Osaka University, 1-8 Yamadaoka, Suita, Osaka, 565-0871, Japan

## Abstract

Osteoblastic mineralization occurs during the early stages of bone formation. During this mineralization, hydroxyapatite (HA), a major component of bone, is synthesized, generating hard tissue. Many of the mechanisms driving biomineralization remain unclear because the traditional biochemical assays used to investigate them are destructive techniques incompatible with viable cells. To determine the temporal changes in mineralization-related biomolecules at mineralization spots, we performed time-lapse Raman imaging of mouse osteoblasts at a subcellular resolution throughout the mineralization process. Raman imaging enabled us to analyze the dynamics of the related biomolecules at mineralization spots throughout the entire process of mineralization. Here, we stimulated KUSA-A1 cells to differentiate into osteoblasts and conducted time-lapse Raman imaging on them every 4 hours for 24 hours, beginning 5 days after the stimulation. The HA and cytochrome *c* Raman bands were used as markers for osteoblastic mineralization and apoptosis. From the Raman images successfully acquired throughout the mineralization process, we found that β-carotene acts as a biomarker that indicates the initiation of osteoblastic mineralization. A fluctuation of cytochrome *c* concentration, which indicates cell apoptosis, was also observed during mineralization. We expect time-lapse Raman imaging to help us to further elucidate osteoblastic mineralization mechanisms that have previously been unobservable.

Osteoblastic mineralization is an important process during the initial stages of bone formation. During mineralization, hydroxyapatite (HA, Ca_10_(PO_4_)_6_(OH)_2_), the major component of bone, is produced, allowing the formation of hard tissue. Previous studies on the process of mineralization have provided valuable insights for the orthopedic and craniofacial dental fields. Although prior studies have revealed that various biomolecules, including type I collagen, alkaline phosphatase, osteopontin, and osteocalcin, are involved in the process of osteoblastic mineralization^1^,^2^,^3^,^4^,^5^,^6^,^7^,^8^,^9^,^10^, the mechanisms directing mineralization are not yet fully understood. In addition, whether the distributions of the various mineralization-related molecules are regulated tightly or occur at random is also unclear. The major obstacle preventing the further elucidation of the process of osteoblastic mineralization is the lack of time-lapse analytical techniques suitable for their study. Traditional biological assays (e.g. polymerase chain reaction analysis and immunostaining) are destructive techniques that cannot be repeatedly applied to observe the time-dependent developments within a single specimen. In order to identify the mineralization mechanisms with precise resolution of the related biomolecules in both time and space, it is necessary to develop a noninvasive analytical technique with subcellular resolution to make time-lapse observations during osteoblastic mineralization.

Osteoblastic mineralization begins with the differentiation of mesenchymal stem cells (MSCs) into osteoblasts. This process is triggered by various types of stimuli, such as cytokines, hormones, or changes in the composition of the extracellular matrix. After the differentiation process initiates, the MSCs differentiate first into osteoprogenitor cells, then into preosteoblasts, and finally into osteoblasts^11^,^12^,^13^. Osteoblasts are the cells that drive the process of mineralization by producing bone matrix proteins, such as type I collagen, osteopontin, and osteocalcin, and form bone tissues by precipitating HA together with these bone matrix proteins^1^,^2^,^3^,^4^,^5^,^6^,^7^,^8^,^9^,^10^,^11^,^12^,^13^. After mineralization, about 60–80% of the osteoblasts die through apoptosis. The remaining osteoblasts further differentiate into osteocytes or bone lining cells. The balance between differentiation and apoptosis plays an important role in maintaining tissue homeostasis and protecting the tissue from serious damage.

Mitochondria play an important role in the intrinsic pathway of apoptosis^14^. The state of the outer mitochondrial membrane is a critical factor that determines whether the cell enters apoptosis or not. The membrane state is regulated by the Bcl-2 family of proteins, which are composed of pro-apoptotic and anti-apoptotic molecular groups. When pro-apoptotic signals overwhelm the survival signals, the function of the anti-apoptotic Bcl-2 proteins is inhibited. As a result, the outer mitochondrial membrane permeability increases, evoking the release of cytochrome *c* from the mitochondrial intermembrane space into the cytoplasmic matrix. Because the release of cytochrome *c* occurs early in the apoptotic process, the altered localization of cytochrome *c* is an indicator of the initiation of the apoptosis pathways.

Currently, most studies on cytochrome *c* use fluorescent labeling to examine the dynamics of cytochrome *c* during apoptosis. However, such labeling techniques have several drawbacks^15^,^16^. First, the fluorescent dye can alter the behavior of the tagged molecule because the size and weight of the dye is often larger than the target molecule. Second, all labeling methods, including transgenic fluorescent expression and immunostaining, require pretreatment of the specimen, which can introduce perturbations to the physiological state of the specimen. Furthermore, photobleaching of the fluorescent signal prevents quantitation and makes fluorescence imaging unsuitable for time-lapse analysis of living cells. Raman microscopy, which can overcome these problems with fluorescence, has been used to study biological processes such as the release of cytochrome *c* during apoptosis because it enables the direct detection of the molecules of interest without any staining^15^.

Raman spectroscopy identifies the chemical signatures of biomolecules in living cells by detecting the scattered light that undergoes frequency shifts compared with the incident light. The magnitude of the frequency shift corresponds to the vibrational energy levels of the biomolecule, which reflects their structure and the surrounding environment. Because Raman spectroscopy simply detects the scattered light from a sample, it is a nondestructive and label-free technique, is optimum for monitoring biochemical processes in living cells^15^,^16^,^17^,^18^,^19^,^20^,^21^,^22^,^23^,^24^. Using a combination of Raman spectroscopy and optical microscopy, we determined the chemical signatures of the biomolecules at different places within the sample tissue^15^,^22^,^23^,^24^.

Although the intensity of spontaneous Raman scattering from sample molecules is typically weak because only a tiny amount of scattered photons, approximately 1 in 10 million, has a different frequency than the incident light, when the frequency of the incident light is close to the electronic transition energy of a specific molecule, the Raman scattering intensity from that molecular species greatly increases. This phenomenon, known as resonance Raman scattering, has an intensity about 10^3^–10^5^ times larger than the spontaneous Raman intensity. The observation of various biomolecules in cells using the resonance Raman has been previously reported^15^,^22^,^23^,^24^. One example is the Raman imaging of cytochrome *c*, which plays an important role in the apoptosis pathways and absorbs light at about 510–550 nm. Okada *et al.* monitored the release of cytochrome *c* from mitochondria during stimulation of the apoptosis pathways by detecting the resonance Raman signal from cytochrome *c* at an excitation wavelength of 532 nm.

Previously, McElderry *et al.* reported Raman spectroscopic study to follow up the mineralization process^21^. In their work, they have used the sophisticated time-resolved Raman measurement system to analyze the mineralization process at the tissue level for several days in several specimens simultaneously with 1 hour time resolution. Meanwhile, we demonstrated that mineralized spots in cultured osteoblasts were visualized by imaging of the 960 cm^−1^ HA Raman band^24^. In the present study, we obtained Raman images of mature KUSA-A1 osteoblasts every 4 hours during osteoblastic mineralization to assess the sequential processes that occur during mineralization. We created time-lapse visualizations of the mineralization process and apoptosis after osteoblastic mineralization by monitoring Raman signals as biomarkers, with HA indicating mineralization and cytochrome *c* showing apoptosis. In addition, we were able to detect β-carotene, a biomolecule that may be an important biomarker for the initiation of biomineralization.

## Results and Discussion

### Construction of time-lapse Raman images obtained from the same osteoblast tissue

**Figure 1:**
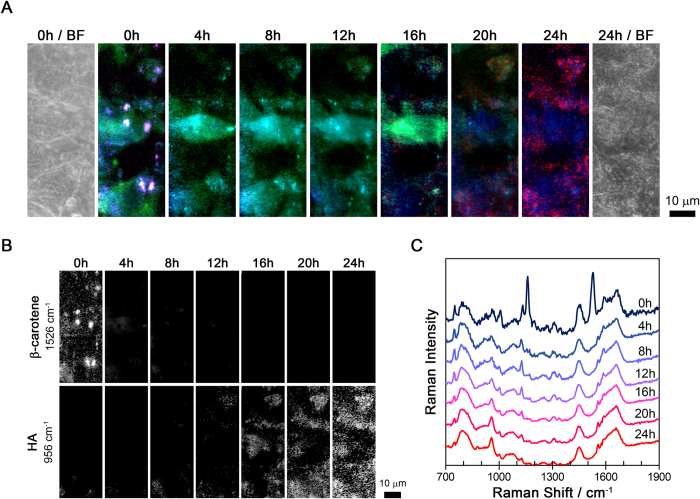
Time-lapse Raman analysis of the same osteoblast tissue. (**A**) Bright-field and time-lapse Raman images of the osteoblasts. The Raman images show cytochrome *c* (750 cm^−1^) as green, HA (956 cm^−1^) as red, β-carotene (1526 cm^−1^) as magenta, and the CH_3_ stretching mode (2940 cm^−1^) as blue. (**B**) The individual time-lapse Raman images for β-carotene and HA. (**C**) The temporal changes in the averaged Raman spectra obtained from the same location in the osteoblasts. Each spectrum was averaged from 234 pixels (about 24 μm^2^).

We performed time-lapse Raman imaging on KUSA-A1 cells every 4 hours starting 5 days after inducing differentiation (i.e. at 120 to 144 hours). We chose day 5 as the starting point because that was the time required for the KUSA-A1 cells to differentiate into osteoblasts and initiate the process of mineralization. In the following text, we define the measurement start time, which was 120 hours after the induction of differentiation, as time zero (0 h). The time-lapse Raman and bright-field images obtained from the same fields-of-view are shown in Fig. 1A. The colors in the Raman images were defined by the 750 (green), 956 (red), 1526 (magenta), and 2940 (blue) cm^−1^ Raman bands, which was assigned to cytochrome *c*, HA, β-carotene, and the CH_3_ stretching mode, respectively^15^,^23^,^24^,^25^,^26^,^27^,^28^,^29^,^30^. The individual time-lapse Raman images of β-carotene (1526 cm^−1^) and HA (956 cm^−1^) are shown in the upper and lower rows of Fig. 1B. The time series Raman spectra from the same osteoblasts are shown in Fig. 1C, as well as the averaged spectra, where the different line colors indicate the different incubation times.

### Interaction between HA and β-carotene in osteoblastic mineralization process

The interesting behavior of β-carotene and HA immediately caught our attention. In our measurement conditions, because the Raman bands of β-carotene were enhanced by the resonance Raman, the location of β-carotene were visualized by Raman imaging. As shown in Fig. 1, the β-carotene Raman bands at 960, 1008, 1160, and 1526 cm^−1^ were seen only at 0 h. A similar pattern was seen when we reconstructed the β-carotene Raman images with the 1526 cm^−1^ Raman band. β-Carotene was localized in several localized spots at 0 h, but not at other time points, as shown in Fig. 1A. In contrast, the Raman signal at 956 cm^−1^, which is assigned to the symmetric stretching of phosphate groups in HA, was not observed at 0 h. After 12 h, the intensity of the HA Raman signal started to increase and the distribution of the HA signal gradually spread out over time. It is easy to confuse the Raman signal around 965 cm^−1^, which appears between 0 and 12 h in Fig. 1C, with the 956 cm^−1^ HA signal. While the difference in their Raman shift was already large enough to conclude that they were of different origins. We defined the 965 cm^−1^ Raman band as octacalcium phosphate (OCP)^31^,^32^ and tricalcium phosphate (TCP)^33^. It is reasonable that the Raman band of OCP and TCP appeared before the HA Raman band because OCP and TCP are precursors to HA^34^,^35^.

It is worth noting that HA appeared to be produced near the spots where β-carotene was localized. Previous studies have reported that β-carotene stimulates the differentiation of osteoblasts by inhibiting cell proliferation and increasing alkaline phosphatase (ALP) activity and the mRNA expression of osteopontin, a matrix protein produced by osteoblasts^36^. β-Carotene can also be converted into retinol, one of the major forms of vitamin A, which is necessary for bone growth, differentiation, and function^36^,^37^,^38^,^39^. Together, our results and those of the previous studies strongly suggest that β-carotene is a biomarker for the initiation of biomineralization.

**Figure 2:**
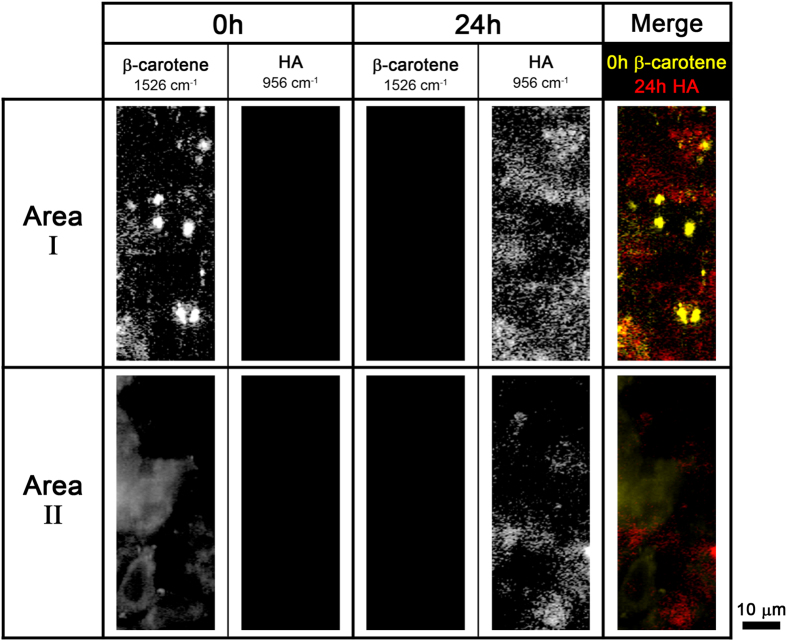
Time-lapse Raman images of β-carotene (1526 cm^−1^) and HA (956 cm^−1^) in two different areas of the osteoblasts. Merged images of 0 h β-carotene (yellow) and 24 h HA (red) Raman images are shown in the last column.

The Raman images of β-carotene and HA at 0 h and 24 h, as well as the merged Raman images of 0 h β-carotene (yellow) and 24 h HA (red) in two different imaging areas are shown in Fig. 2. The β-carotene Raman signal in area I was stronger and more widely distributed than that in area II. This result indicates that more β-carotene was contained in area I than in area II because the spectral intensity is directly proportional to the concentration of the molecules. Similar to β-carotene, HA was more abundant and widely distributed in area I than in area II. This result suggests that the degree of osteoblastic mineralization is proportional to the accumulated β-carotene concentration. β-Carotene increases ALP activity and promotes the expression of osteopontin in a dose-dependent manner^36^. In addition, HA appeared to be localized close to where β-carotene was previously found in both areas. The data in Fig. 2 also support our hypothesis that β-carotene is a biomarker for the initial stages of mineralization.

### Chronological changes in mitochondrial cytochrome *c* of the osteoblasts

**Figure 3:**
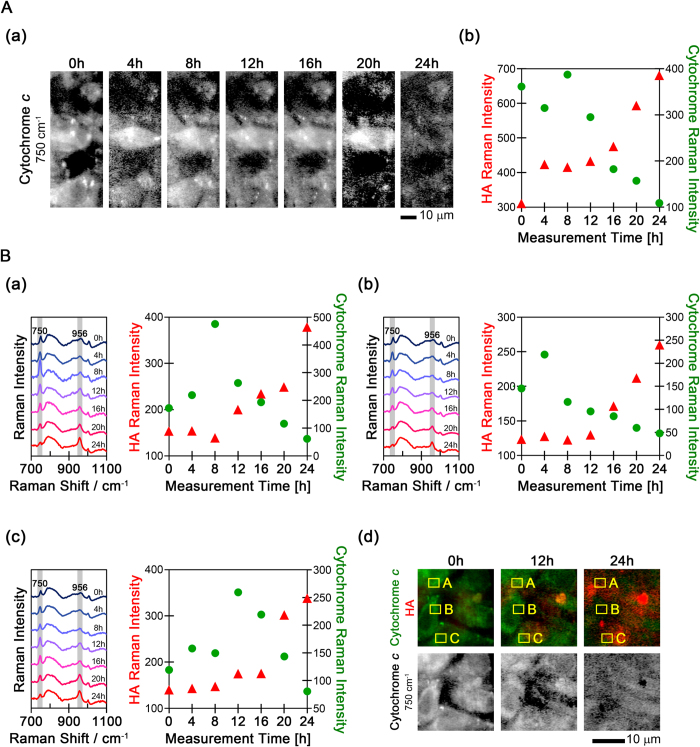
The temporal changes of cytochrome *c* and HA Raman signals from osteoblasts. (**A**) The temporal changes in cytochrome *c* are shown as (a) Raman images and (b) Raman intensity plots. The Raman intensity of HA is also shown in (b). (**B**) The temporal changes in cytochrome *c* and HA Raman intensity in three additional locations Panels (a–c) indicate the temporal changes in the averaged Raman spectra and Raman intensity of cytochrome *c* and HA in the three cells. The averaged Raman spectra were obtained from the regions boxed in yellow labeled A, B, and C in (d). Panel (d) shows the time-lapse merged Raman images of cytochrome *c* (750 cm^−1^, shown in green) and HA (956 cm^−1^, shown in red) and the individual Raman images of cytochrome *c*.

Next, we determined the changes in cytochrome *c* using time-lapse Raman analysis. Strong Raman peaks from cytochrome *c* were found near 750, 1126, 1310, and 1589 cm^−1^. The Raman images of cytochrome *c* were reconstructed using the Raman band at 750 cm^−1^ assigned to the pyrrole breathing mode ν_15_ in cytochrome *c*. The Raman signal of cytochrome *c* decreased over time after 16 h, along with an increase in the HA peak, as shown in Fig. 3A. This reduction of the cytochrome *c* signal may be caused by apoptosis. During the early stage of apoptosis, the concentration of cytochrome *c* decreases because cytochrome *c* is released from the mitochondria into the cytosol to trigger the caspase activation cascades. In addition, only oxidized cytochrome *c* induces caspase activation via the formation of apoptosomes, and the Raman signal from oxidized cytochrome *c* is too weak to be detected under biological conditions. Cytosolic cytochrome *c* in apoptotic cells is rapidly oxidized by mitochondrial cytochrome oxidase, whereas cytosolic cytochrome *c* in healthy cells is rapidly reduced by various enzymes and/or reductants^15^,^40^.

The intensity changes of the HA and cytochrome *c* peaks in the Raman spectra are shown in Fig. 3A(b). After 12 h, the intensity of the cytochrome *c* Raman bands decreased with time, while the HA band intensity increased. And the strongest cytochrome *c* peak was observed just before the decrease, in this case at 8 h. To determine whether this is a general phenomenon or not, the intensity changes of the HA and cytochrome *c* peaks from three different cells were plotted in Fig. 3B(a–d). In all the images, the HA signals are labeled in red and the cytochrome *c* signal in green. As shown in Fig. 3B(d), the increase in HA intensity and decrease in cytochrome *c* intensity was observed throughout the mineralization process. And we observed the intensity peak of the cytochrome *c* signal at different times in each cell: at 8 h in cell A, at 4 h in cell B, and at 12 h in cell C. As seen in Fig. 1A, the timing of the strongest cytochrome *c* signal appeared different in each cell. Prior work has shown that mitochondrial cytochrome *c* enrichment is one of the early events that precede the onset of apoptosis and that the increase of mitochondrial cytochrome *c* is observed before the release of cytochrome *c* into the cytosol^41^,^42^. Thus, the increase of cytochrome *c* likely indicates the start of apoptosis. Our results suggest that Raman spectral imaging detects the start of apoptosis occurring at different times in different cells.

## Conclusions

**Figure 4:**
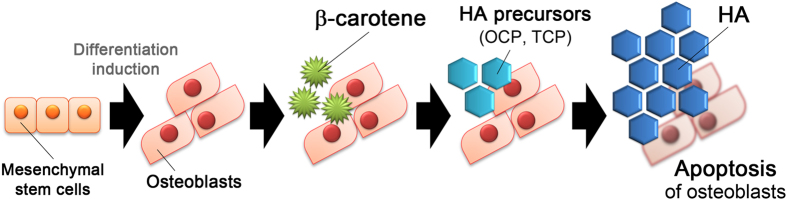
Schematic illustration of our hypothesis based on the results of the present study. First, β-carotene is localized around the initiation sites of mineralization in osteoblasts, and then HA precursors, including OCP and TCP, are produced near the sites where β-carotene was located. After the production of the HA precursors, HA seeds are generated and mature into hard tissues. At the same time, apoptosis of the osteoblasts is initiated and eventually the osteoblasts die.

We report here the longitudinal time-lapse Raman imaging analysis of individual osteoblasts throughout the process of mineralization. Our results suggest that β-carotene was used as a biomarker to indicate the initiation of osteoblastic mineralization. During mineralization, most of the HA was produced near the spots where β-carotene was previously localized. In addition, the Raman images suggested that the more concentrated the β-carotene was, the more HA was produced in the surrounding areas. Although many studies have been published on how β-carotene promotes mineralization, none of them have identified β-carotene as an intrinsic marker of the initiation of mineralization. Our present study is the first to suggest that β-carotene could be used in this way. The time-lapse Raman imaging also showed the changes in the distribution of cytochrome *c* during the apoptosis of the osteoblasts. Temporal increases in cytochrome *c*, an early indicator of apoptosis, were observed just prior to decreases in cytochrome *c* concentration. Our hypothesis for one mechanism of mineralization based on the results of this study is shown in Fig. 4. First, β-carotene, an agent that promotes osteoblastic differentiation and mineralization, accumulates at the initial mineralization sites after the KUSA-A1 cells have differentiated into osteoblasts. Next, HA precursors including OCP and TCP are produced near the areas where β-carotene existed. Through the production of HA precursors, HA seed crystals are generated and mature into hard tissues by taking Ca^2+^ and PO_4_^3−^ from the extracellular fluid. At the same time, the process of apoptosis is initiated in the osteoblasts, which eventually die. These details could not have been revealed without using Raman imaging techniques. Time-lapse Raman imaging provided the dynamic localization and concentration information about these biomolecules related to osteoblastic mineralization at a subcellular resolution. In the future, we plan to apply noninvasive optical techniques such as Raman imaging to the study of the biological processes of hard tissue formation with more precision to better understand these processes at the molecular level.

## Methods

### Cell culture

Osteoblasts were obtained by differentiating KUSA-A1 cells, a mouse mesenchymal stem cell line derived from the C3H/He mouse strain (Riken Cell Bank, Tsukuba, Japan). KUSA-A1 cells were cultured at 37 °C in a humidified atmosphere with 5% CO_2_ in culture medium. The culture medium was alpha minimum essential medium (α-MEM; CM1401-012, Nikken Bio, Kuze, Japan) supplemented with 10% fetal calf serum (FCS; FBS01-500, Biofill Australia Pty. Ltd., Elsternwick, Australia) and 60 mg/L kanamycin sulfate (119-00703, Wako, Osaka, Japan). These cells were plated at about 2,500 cells/cm^2^ on a 100 mm TC-treated culture dish (430167, Corning Life Sciences, Tewksbury, USA). For subculturing, the cultured cells were recovered from the substrate 3 days after the last plating and replated at the same density.

To differentiate the KUSA-A1 cells into osteoblasts, osteogenic differentiation medium, α-MEM supplemented with 10% FCS, 60 mg/L kanamycin, 10 mM β-glycerophosphate disodium salt hydrate (G9422, Sigma-Aldrich, St. Louis, USA), and 50 μg/mL L-ascorbic acid phosphate magnesium salt n-hydrate (013-12061, Wako) was used as the culture medium. The differentiation medium was replaced every 3 days.

In this research, cultured KUSA-A1 cells, a mouse mesenchymal stem cell line, were used and we didn’t use any live vertebrates and/or higher invertebrates.

### Time-lapse Raman imaging

To avoid autofluorescence from tissue culture substrates, the KUSA-A1 cells were cultured and differentiated into osteoblasts on a quartz dish (12 mm in diameter; SF-S-D12, Synapse, Seattle, USA). We first confirmed that the growth rate, morphology, and time required for differentiation of the KUSA-A1 cells were similar when cultured on quartz or glass substrates. Prior to the Raman measurement, the medium was replaced with HEPES-buffered Tyrode’s solution, which comprised 150 mM NaCl, 10 mM glucose, 10 mM HEPES, 4.0 mM NaOH, 4.0 mM KCl, 1.0 mM MgCl_2_, and 1.0 mM CaCl_2_. After each Raman measuring, the medium was immediately changed to osteogenic differentiation medium and the sample cells were cultured at 37 °C in a humidified atmosphere with 5% CO_2_.

All of the Raman spectra and Raman images were obtained with a custom made slit-scanning Raman microscope. The slit-scanning Raman microscope was equipped with a 532 nm Nd:YVO_4_ frequency doubling laser (Verdi, Coherent Inc., Salem, USA) and a 60X/1.27 NA water-immersion objective lens (CFI Plan Apo IR 60XW, Nikon, Chiyoda, Japan). A line-shaped laser beam was formed using cylindrical lens sets and focused on the sample plane by the objective lens. The backscattered Raman signals from the sample were collected by the same objective lens and arrived at a spectrometer (MK-300, Bunkoh-Keiki Co. Ltd., Hachioji, Japan) after passing through a 532 nm long-pass edge filter (LP03-532RU-25, Semrock, Rochester, NY, USA). The signals were then split into different wavelengths by a grating, and the dispersed light was detected by a cooled CCD camera (Pixis 400B, Princeton Instruments, Acton, USA). The slit width of the spectrometer was set to 50 μm and a 600-grooves/mm grating was used. The quartz dish was positioned on the microscope stage for the measurement, and the line-shaped laser was scanned with a single-axis galvanometer mirror (710–745825, 000-3014016, GSI Lumonics, Bedford, USA). The Raman spectra and images were taken every 4 hours for 24 hours, a total of 7 times. The pitch of the line scan was 500 nm. The excitation laser intensity at the sample plane was 1.5 mW/μm^2^, and the exposure time for each line was 1.5 s.

Prior to the Raman image reconstruction, a singular value decomposition (SVD) was performed to reduce noise in the obtained spectra^43^. As shown in Figure S1, in the first seven components some Raman spectral information were observed, while from the eighth component the noise was dominant. Therefore we manually chose the first seven or eight components depending on datasets to reconstruct the image so that the image contrast is considerably enhanced. After the SVD calculation, the fluorescence background signal was subtracted from the Raman spectra at each pixel in the image using a modified polynomial fitting technique^23^,^44^.

Raman images of Figs 1(A,B) and 2-Area I, and Fig. 3(A) were measured on the same culture. And Raman images of Fig. 2-Area II and Fig. 3(B) were also measured on the same culture which was different from the foregoing culture. The averaged Raman spectra were obtained from 234 pixels (about 24 μm^2^) in Fig. 1C and from 91 pixels (about 9.5 μm^2^) in Fig. 3B.

## Additional Information

**How to cite this article**: Hashimoto, A. *et al.* Time-lapse Raman imaging of osteoblast differentiation. *Sci. Rep.* **5**, 12529; doi: 10.1038/srep12529 (2015).

## Supplementary Material

Supplementary Information

Supplementary Information
