# Supplementary material for: Time-lapse Raman imaging of osteoblast differentiation
Source: Sci Rep. 2015 Jul 27;5:12529. doi: 10.1038/srep12529 (PMC4515588; doi:10.1038/srep12529)
Supplement: Supplementary Information [file srep12529-s1.pdf]

Time-lapse Raman imaging of osteoblast differentiation

Supplementary Information

Aya Hashimoto[a], Yoshinori Yamaguchi[a,b,*], Liang-da Chiu[a], Chiaki Morimoto[c],

Katsumasa Fujita[a], Masahide Takedachi[c], , Satoshi Kawata[a],

Shinya Murakami[c], and Eiichi Tamiya[a,*]

[a] Department of Applied Physics, Graduate School of Engineering, Osaka University, 2-1 Yamadaoka, Suita, Osaka, 565-0871, Japan

[b] Department of Physics, Graduate School of Science, East China University of Science and Technology, 130 Meilong Rd., Shanghai, 200237, China

[c] Department of Periodontology, Graduate School of Dentistry, Osaka University, 1-8 Yamadaoka, Suita, Osaka, 565-0871, Japan

*Corresponding authors:

Yoshinori Yamaguchi, yoshi.yamaguchi@ap.eng.osaka-u.ac.jp

Eiichi Tamiya, tamiya@ap.eng.osaka-u.ac.jp

Figure Legends

Figure S1. Noise reduction of Raman spectra by SVD calculation. (A) Raw and processed data of Raman spectra obtained from KUSA-A1. (B) Plots of the first ten basis vectors resulting from SVD calculation. The blue-letter values indicate contribution factor of the basis vectors.
