# Supplementary figures and images for: Time-lapse Raman imaging of osteoblast differentiation
Source: Sci Rep. 2015 Jul 27;5:12529. doi: 10.1038/srep12529 (PMC4515588; doi:10.1038/srep12529)

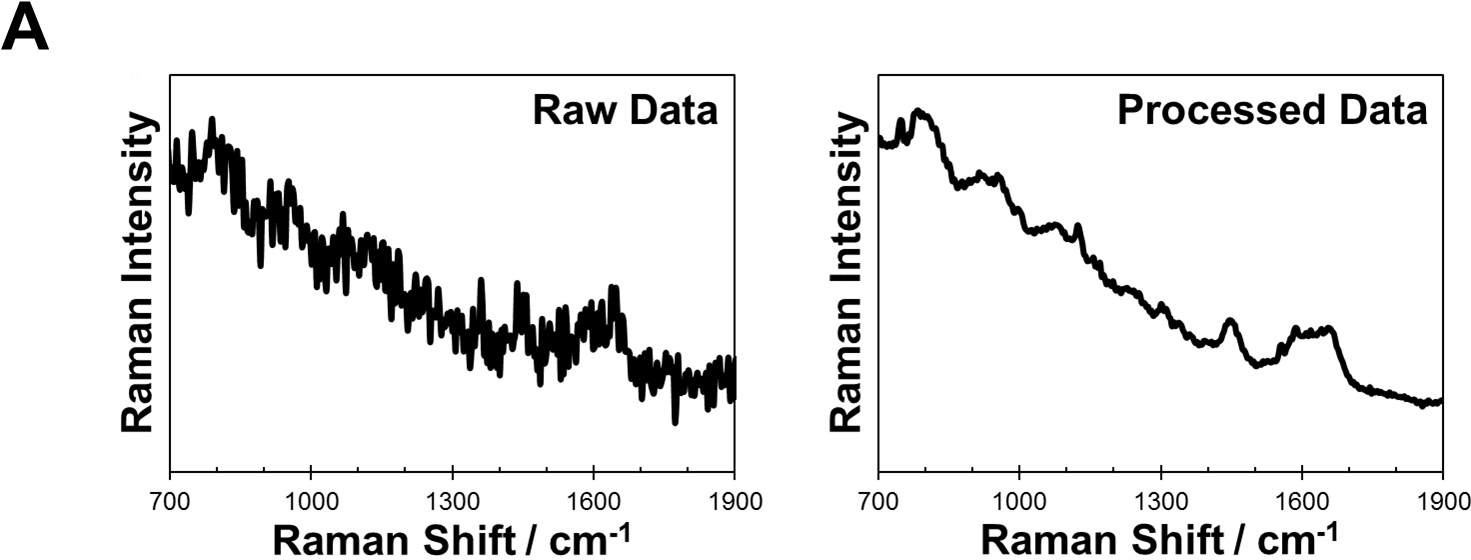


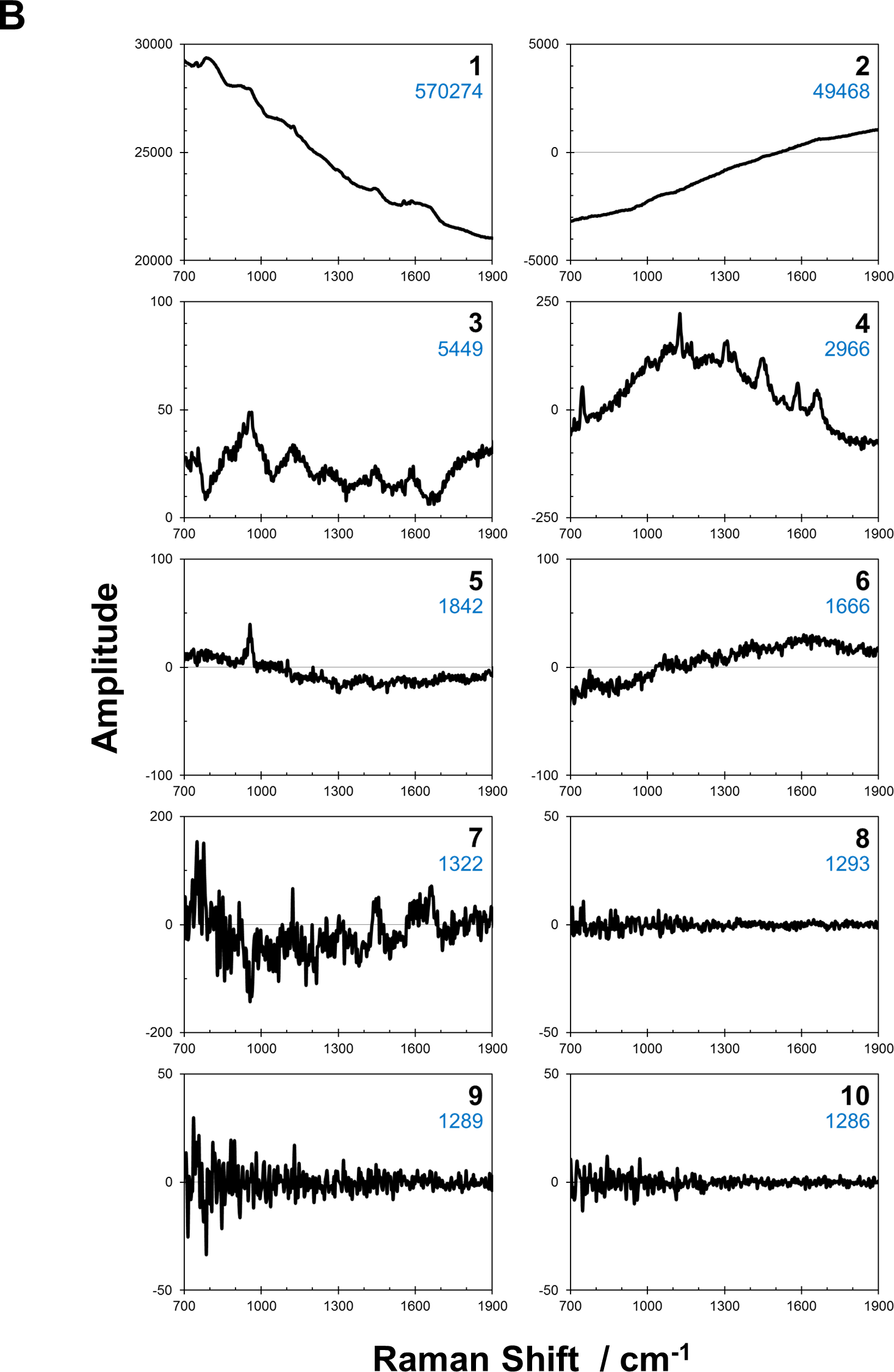

Supplement: Supplementary Information [file srep12529-s2.doc]
